# Avian Metapneumovirus: Virology, Epidemiology, and Insights from a Comparative Analysis with Human Metapneumovirus—A Review

**DOI:** 10.3390/biom16030351

**Published:** 2026-02-26

**Authors:** Jason S. Hatfield, Beth K. Thielen, Sagar M. Goyal

**Affiliations:** 1 Veterinary Population Medicine Department and Veterinary Diagnostic Laboratory, College of Veterinary Medicine, University of Minnesota, St. Paul, MN 55108, USA; hatfi134@umn.edu; 2 Division of Pediatric Infectious Diseases, Department of Pediatrics, University of Minnesota Medical School, Minneapolis, MN 55455, USA; thie0149@umn.edu

**Keywords:** avian metapneumovirus, human metapneumovirus, molecular virology, vaccines, review

## Abstract

Metapneumoviruses comprise a genus of negative-sense RNA viruses that cause significant respiratory disease across human and avian hosts. Human metapneumovirus (hMPV) is a globally prevalent pathogen associated with acute lower respiratory tract infections in infants, older adults, and immunocompromised individuals. Avian metapneumovirus (aMPV) imposes substantial economic losses on the poultry industry through respiratory disease, reproductive impairment, and high mortality in the presence of secondary infections. Despite their distinctive host ranges, hMPV and aMPV share a conserved genomic architecture and encode homologous structural and non-structural proteins that mediate viral entry, replication, assembly, and evasion of host innate immunity. Comparative analysis highlights that both have deeply conserved polymerase and nucleocapsid functions, and yet have a wide range of diversity in the attachment glycoprotein (G) and small hydrophobic protein (SH), reflecting divergent evolutionary pressures in human versus avian hosts that have led to such distinctive differences. The recent emergence and detection of aMPV/A and aMPV/B across the previously aMPV-free United States beginning in late 2023, combined with rising cases globally of hMPV post-SARS-CoV-2 pandemic, underscore the continued challenges of metapneumovirus surveillance and control in humans and animals. This review aims to highlight the current knowledge on the history, molecular virology, pathogenesis, epidemiology, diagnostics, and control strategies for aMPV while drawing mechanistic parallels to hMPV. By contextualizing shared biology and structure alongside host-specific adaptations, we aim to identify key gaps that shape vaccine design, antiviral development, and future research priorities aimed at mitigating the health and economic burden posed by metapneumoviruses found in both birds and humans.

## 1. Introduction

Metapneumoviruses are small, enveloped, negative-sense single-stranded RNA viruses classified within the family *Pneumoviridae*, genus *Metapneumovirus* [1]. This family was reclassified from within *Paramyxoviridae* in 2016 and currently comprises two genera, *Orthopneumovirus* and *Metapneumovirus*. Members of the *Metapneumovirus* family share a conserved genomic structure and lack the defining NS1/NS2 genes that characterize orthopneumoviruses. The *Metapneumovirus* genus consists of two viruses, human metapneumovirus (hMPV) and avian metapneumovirus (aMPV) [1,2].

The hMPV was first detected in the Netherlands in 2001 and is now recognized as a global cause of acute respiratory infection across all age groups. In healthy adults, infections are mild or subclinical, whereas they cause acute lower respiratory disease in young children, the elderly, and immunocompromised patients. As of writing this review, there are currently no licensed antivirals or vaccines available, and the mainstay treatment is supportive care [3,4,5,6].

The aMPV is a global respiratory virus that was first detected in South Africa in 1978. This virus primarily infects chickens and turkeys [7], causing respiratory distress, reduced weight gain, poor feed conversion, and a marked decline in egg production and shell quality that leads to significant losses to the poultry industry [8,9]. Disease severity often increases due to co-infections with secondary bacterial or viral infections that can lead to high morbidity and mortality. Both live and inactivated vaccines are available in several parts of the world, but antigenic diversity and logistical constraints have complicated eradication and control efforts [10,11].

The emergence of zoonotic diseases with spillover events, such as the COVID-19 pandemic, Lassa Fever, Ebola, Marburg, or the most recent H5N1 influenza outbreak in dairy cattle, highlights the importance of investigating metapneumoviruses in depth [12,13,14,15,16,17]. Beyond their biological similarities, hMPV and aMPV pose distinct but overlapping public health and economic challenges that justify a comparative analysis to develop appropriate intervention strategies and treatments.

This review aims to provide a comprehensive overview of the classification, molecular virology, pathogenesis, epidemiology, diagnostic practices, and control strategies for aMPV, while drawing parallels to hMPV, where relevant. By examining both viruses, we aim to highlight key areas of overlap and divergence that may provide a deeper context for both viruses and inform future research directions and the ongoing development of intervention strategies.

## 2. History

### 2.1. History of Human Metapneumovirus

The hMPV was first isolated and described in 2001 from nasopharyngeal aspirates of young children with respiratory tract infections in the Netherlands [18]. Initial characterization showed that the genomic organization and sequence homology of hMPV closely resembled those of human respiratory syncytial virus (RSV) but were notably missing two nonstructural proteins, NS1 and NS2. This placed the newly discovered hMPV into the *Metapneumovirus* genus, which had never been associated with a disease in humans [19,20]. Follow-up surveillance studies showed that hMPV infects virtually all children by 5–10 years of age and that it could be found in every country around the world [21,22,23,24,25]. Retrospective serological and molecular surveys identified hMPV-like viruses in archived specimens dating back to at least 1958, indicating that hMPV had circulated in human populations long before it was formally recognized [18,21]. These retrospective analyses and epidemiologic studies established that hMPV commonly causes upper and lower respiratory tract infections in infants and young children and contributes substantially to morbidity in elderly adults and immunocompromised individuals [24,26]. The hMPV likely went undiscovered for so long due in part to its similarity to RSV, as they both infect the same demographics, can be found globally, and cause similar clinical symptoms [18,27].

Molecular and antigenic studies have consistently divided hMPV into two major genetic lineages, historically designated genotype A and genotype B [28,29]. Early genotyping identified four principal lineages, A1, A2, B1, and B2, although A1 has not been isolated recently [28,30,31]. Subsequent studies over the years with new technology that provided higher resolution phylogenetic analyses have revealed that the A2 lineage shows the most genetic diversity among the subtypes and that it can actually be divided into four clades, historically labeled: A2a, A2b1, A2b2, and A2c [32,33,34]. However, recent genome-wide phylogenetic analysis has shown that A2c corresponds with A2b2, with both commonly sharing the 111- and 180-nucleotide G-gene duplication variants that originally led to their original distinction as two sublineages. Thus, A2c and A2b2 are now referred to as the same unified monophyletic clade “A2b2” to better support future hMPV surveillance work [33,35]. A competing numerical lineage system, following the nomenclature used for influenza viruses, was introduced in 2022 and has since been adopted by several researchers to describe hMPV lineages [35,36,37,38]. This system divides the A2 lineage and its sublineages from A2, A2a, A2b1, and A2b2 into A2.1, A2.2, A2.2.1, and A2.2.2, respectively, in an attempt to better distinguish different phenotypes and phylogenetic clusters [36]. Neither of them is ratified as the official hMPV nomenclature system, and both are currently in use. Notably, Nextstrain, a popular, open-source, real-time viral evolution tracker that is used in many hMPV phylogeny and genomic analysis studies, has adopted the new numerical lineage system as part of its tracking system [30,31]. Epidemiological sampling over the past two decades indicates that all of these sublineages continue to co-circulate globally. Relative prevalence is variable and can be regionally heterogeneous and change from season to season [30,31,39,40,41,42,43].

### 2.2. History of Avian Metapneumovirus

The aMPV was first identified in turkey flocks in South Africa in 1978 [7]. Initially called “Turkey Rhinotracheitis Virus” (TRT), the virus spread quickly into turkey flocks in Europe and parts of Asia throughout the 1980s. During this time, another new disease, initially referred to as “Swollen Head Syndrome” (SHS), was seen spreading in chickens in the same regions. Studies quickly showed that TRT and SHS were caused by the same virus, first known as “Avian Pneumovirus” (APV), but what we now call modern-day aMPV.

The aMPV is classified into four subtypes (A, B, C, D). The subtypes A and B are most prevalent in Europe, Brazil, and Africa, while subtype C has been reported from the US, Asia, and Europe. Subtype D has been identified only in France from a retrospective analysis of a 1985 outbreak and has not been seen since then [44,45]. Australia is the only continent that is still considered aMPV-free. In all cases, aMPV/B has emerged as the most prevalent subtype compared to aMPV/A [46].

The United States (U.S.) was aMPV-free until aMPV/C was detected in 1996 in Colorado. This subtype spread to multiple surrounding states in the Midwest through the late 1990s and into the early 2000s before a live-attenuated vaccine, combined with stricter biosecurity measures, eradicated the disease from the U.S. by the late 2000s. Any subsequent outbreaks were contained and handled quickly to the point that the U.S. was considered aMPV-free for the last decade until detection of aMPV/A in October 2023 in California, and aMPV/B by December 2023 in turkeys and broilers in several states in the South, including West Virginia, Virginia, Georgia, South Carolina, and North Carolina, along with detection of aMPV-A in Texas and California [11,47,48]. In less than six months from its first detection, aMVP/A and aMPV/B spread rapidly throughout the U.S. and were detected in 26 states and into two Canadian provinces. As of the time of writing this review in November 2025, the aMPV outbreak in the U.S. remains uncontrolled and widespread across multiple states, with ongoing detection and regulatory emergency actions to help farmers affected by the spread of aMPV [11,49].

## 3. Viral Genome

### 3.1. Metapneumovirus Viral Genome

**Figure 1:**
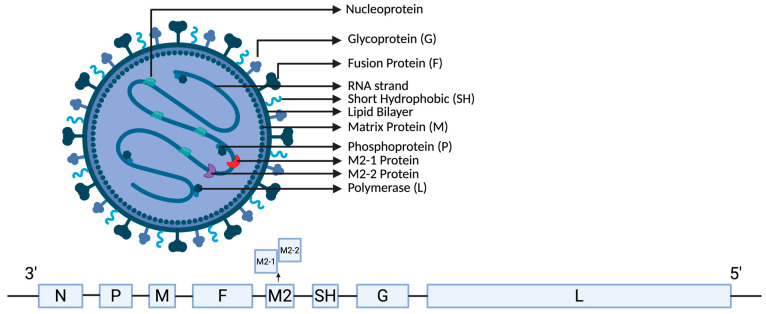
The genome structure of hMPV and aMPV was created with BioRender.com. Hatfield, J. (2026) https://BioRender.com/f0mgao2.

The genomes of both aMPV and hMPV are ~13 kb in length and have similar genomes in the same gene order, encoding eight major genes ordered 3′-N-P-M-F-M2-Sh-G-L-5′ (Figure 1). These eight genes encode nine proteins, which include: nucleoprotein (N), phosphoprotein (P), matrix protein (M), fusion glycoprotein (F), two overlapping matrix 2 proteins (M2-1 and M2-2), small hydrophobic protein (SH), attachment glycoprotein (G), and large RNA-dependent RNA polymerase (L) [50]. Table 1 provides a summary of the comparisons for hMPV and aMPV viral genomes.

### 3.2. N Protein

The N protein encapsidates the negative-sense genomic RNA and forms the ribonucleoprotein complex (RNP) with P and L, which is the template for transcription and replication [51,52]. N binds to RNA with high affinity, oligomerizes along the genome, and provides the structural scaffold required for polymerase engagement from P and L [53,54]. This interaction protects viral RNA from nuclease activity and presents the genome in a correct conformation to the viral polymerase [44,51,53]. The N protein is highly conserved between aMPV and hMPV, sharing approximately 70–99% of amino-acid identity, with some aMPV/C and hMPV strains being nearly identical in sequence [44,50].

### 3.3. P Protein

The P protein is the polymerase cofactor that bridges N-bound RNA and L polymerase to the nucleocapsid. It chaperones newly synthesized N in its monomeric form to prevent non-productive RNA binding and co-localizes with actin in host tissues to induce the formation of cellular extensions that increase cell-to-cell spread [50,51,52,54,55]. P has a central role in coordinating enzymatic activities of the polymerase complex, as it promotes phase separation with N and drives inclusion body formation by organizing replication compartments through multivalent N-P interactions [54,56,57,58]. P is moderately conserved between metapneumoviruses, with aMPV/C sharing approximately 67–68% of its amino-acid identity with the different hMPV subgroups and around 53–56% of its amino acid identity with other aMPV strains [50].

### 3.4. M Protein

The M protein is a multifunctional structural protein that lines the inner viral membrane and is essential for virion assembly and budding by linking the RNP to budding sites [59]. It contains a YSKL and a YAGL motif, which are important determinants for viral assembly and spread [60]. Structural analysis of M shows that it contains a dimeric assembly and has high-affinity Ca^2+^ binding sites that stabilize the viral folds and oligomerization, aiding in host cell budding efficiency and infection [59,61]. Extracellular M can be taken up by dendritic cells and macrophages, thereby activating them and inducing proinflammatory cytokines, which may contribute to the strong inflammatory response seen in hMPV infections [62]. The M protein of aMPV likely plays an analogous role in virion structure, though the specific immunomodulation functions in avian hosts have not been as well characterized as in humans. Comparative analysis shows high sequence conservation among all aMPV and hMPV subtypes, with 87–88% between aMPV/C and hMPV, 78–79% between aMPV/C and aMPV/A or aMPV/B, and all other subtypes sharing between 90–99% between each other for both hMPV and aMPV strains [44].

### 3.5. F Protein

**Figure 2:**
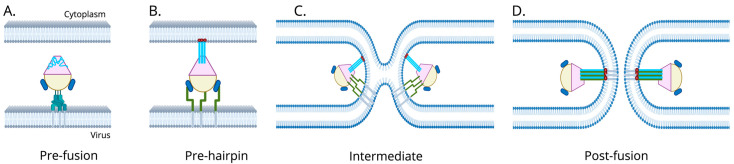
Metapneumovirus fusion model of the F protein. (**A**) The F protein is displayed on the virion surface in a metastable pre-fusion conformation. The HRA (blue) is constrained in this state, while the HRB (green) forms a stalk-like connection to the transmembrane domain. (**B**) Following activation, extensive refolding of the heptad repeat domains occurs, during which HRA extends into a trimeric coiled-coil and the hydrophobic fusion peptides (red) are exposed and inserted into the target cell membrane, generating an elongated fusion intermediate. (**C**) These intermediate bridges the viral and cellular membranes, with multiple F trimers acting together to draw the two lipid bilayers into close proximity. The unstable nature of this state facilitates membrane deformation and alignment. (**D**) Final folding of HRB back against the HRA core results in the formation of the six-helix bundle (6-HB), composed of three antiparallel HRB helices packed around the HRA trimer. Completion of this structure brings the viral and cellular membranes together, leading to membrane merger and fusion pore formation [74,75]. Created in BioRender.com. Hatfield, J. (2026) https://BioRender.com/mf1spz7.

The F protein is a class I fusion protein that mediates virus-cell membrane fusion and entry of the virus [63]. It is the principal target of neutralizing antibodies from the host immune system and is a major target for vaccine and drug development [63,64]. The F protein is trimeric and must be proteolytically cleaved to become fusogenic, which is typically done by host proteases [63]. Both hMPV and aMPV exploit host integrins for cell entry, although they engage different motifs. F undergoes a conformational change upon triggering, delivering the genome into host cells [65,66]. The mechanism of membrane fusion by the F protein is illustrated in Figure 2. This understanding is best described in hMPV, which is often shared in RSV research as they have similar F protein structure and function [67]. The hMPV F protein is assembled as a trimer in a metastable pre-fusion conformation. Upon viral attachment to the host cell, the F protein is activated and undergoes a series of conformational changes to initiate fusion of the viral and cell membranes [68]. These structural changes convert F from its high-energy pre-fusion state into a highly stable post-fusion conformation. Proteolytic cleavage of F generates the F1 subunit, which contains the N-terminal hydrophobic fusion peptide and two hydrophilic regions that are conserved hepta-repeats (HR). This includes the N-terminal heptad (HRA), which is part of the pre-fusion head as short α-helices connect by loops, and the C-terminal heptad (HRB), which connects the globular head of the pre-fusion viral membrane to the transmembrane region via a stalk of three α-helices [69,70,71,72]. Following activation, the fusion peptide is projected outward and inserts into the host cell membrane, forming an extended intermediate that bridges the viral and cellular membranes. During this stage, HRA refolds into a long trimeric coiled-coil, creating a central scaffold often referred to as the pre-hairpin intermediate. Subsequent refolding of HRB back along the HRA core results in the formation of a six-helix bundle (6-HB), composed of three antiparallel HRB helices packed against the internal HRA trimer. Once 6-HB is complete, formation of the fusion pore occurs, which allows the delivery of the viral nucleocapsid into the cell cytoplasm to initiate a new infection cycle [69,73].

The hMPV F protein contains an Arginine-Glycine-Aspartic acid (RGD) motif in its head region that binds to RGD-specific integrins like αVβ1, αVβ5, and αVβ8 on human airway cells, facilitating viral attachment and entry [65,76]. aMPV contains 2 different motifs that are subtype-strain dependent. aMPV/B contains an Arginine-Aspartic Acid-Aspartic Acid (RDD) motif that engages aVB1, while aMPV/C contains an Arginine-Serine-Aspartic Acid (RSD) motif that binds to integrin B1 (ITGB1) [77,78]. Both aMPV and hMPV require different environments for fusion to occur; hMPV requires a low pH, while aMPV does not [66,79]. F is highly conserved among all hMPV sublineages at 81–82% and decently conserved between aMPV/A and aMPV/B at 71–73%. aMPV/C has less overlap with other aMPV strains at 39% but shares a higher identity with hMPV strains at 56–61% [44].

### 3.6. M2 Protein

The M2 gene encodes two overlapping open reading frames, M2-1 and M2-2, which have distinct roles in viral RNA synthesis [50,80]. M2-1 functions as a transcriptional elongation factor and antitermination protein that interacts with polymerase transcription and enhances the synthesis of readthrough mRNAs [80]. It contains a zinc-binding CCCH motif required for activity and is essential for viral transcription and replication in vivo [81,82]. M2-1 is well conserved between aMPV and hMPV, sharing approximately 52–70% amino acid identity [83]. In contrast to M2-1, M2-2 is a small regulatory protein that inhibits host transcription and genome replication [84]. It plays a major role in regulating miRNA expression and can inhibit RIG-I/TRIM25 signaling, disrupting interferon (IFN)-β activation in host cells [85,86]. A loss or mutation of M2-2 alters the ratio of mRNA synthesis and genome replication and reduces viral fitness, which would support a hypothesis that it is important for host-specific immune evasion [87]. Sequence conservation of M2-2 between aMPV and hMPV is substantially lower than that in M2-1, with less than 20% of shared amino acid identity [44,83].

### 3.7. SH Protein

The SH gene encodes a tiny membrane-embedded glycoprotein whose exact functions are not fully understood. Studies show that it has viroporin effects that inhibit NF-κB transcriptional activity and may perform a modulatory role in fusogenic activity, regulating fusion protein function and membrane permeability during viral infection [88,89,90]. Recent studies have shown that it inhibits interferon induction in plasmacytoid dendritic cells and can interfere with host immune signaling by promoting degradation of the host kinase Jak1, which dampens IL-6 and interferon responses [91,92]. Despite these functions, SH is nonessential in viral replication in many cell culture systems. Deletion of SH does not impact viral replication but can reduce viral growth rates [92,93]. The sequence conservation amongst SH proteins varies wildly between aMPV subtypes, with conservation as low as 42–49% between aMPV/A, aMPV/B, and aMPV/D [44]. Strains of aMPV/C have been reported as having as low as 10–12% shared amino acid identity when compared to aMPV/A or aMPV/B [94]. When compared to hMPV, all aMPV subtypes shared between 14–31% amino acid identity, with aMPV/C being the closest to hMPV [44,50]. This divergence and diversity suggest that while SH may share a broad archetype across species in terms of its location in the genome, size, and general functions, the specific host-interaction motifs vary considerably even among viral subtypes. More research is needed to better understand the interactions of SH with the rest of the genome to understand the differences for each subtype of aMPV and hMPV.

### 3.8. G Protein

The G gene encodes an attachment glycoprotein on the virus surface that contributes to host–cell binding and evasion of the host cell’s innate immune response [95,96,97]. It is considered an accessory attachment protein that modulates but is not always essential for viral entry. Thus, the F protein can mediate attachment and entry when G is removed [98,99]. The deletion of G does not impact the replication ability of the virus, but can slow its growth [93]. The G protein is heavily glycosylated and displays high variations in length, glycosylation sites, and sequence between genetic lineages within species for both hMPV and aMPV, and is the most divergent metapneumovirus protein between host species [38,100,101]. There is very low shared amino-acid identity between hMPV and aMPV G sequences, ranging from 20–30% depending on which aMPV subtype is compared to which hMPV subtype [102,103]. Comparisons within lineages of each virus can vary widely, as hMPV has been shown to have a shared sequence identity as low as 29% between subtype A and B, with an average of 63%, and aMPV showing as low as 14–16% amino acid identity between subgroups [100,103]. In addition to point mutations and glycosylation variability, several independent partial duplication events in the hMPV G gene have been identified epidemiologically and are associated with lineage expansion as early as 2014 [39,104,105]. Predominant duplication types include 111-nt, which is mostly found in the A2 clade, and 180-nt insertions, which are mostly found in the B2 clade. The 180-nt insertion results in a corresponding 60-amino-acid duplication in the G protein [104,105,106]. This expanded region is predicted to introduce numerous additional potential O-linked glycosylation sites, further increasing the already extensive glycan shielding of G [104]. Phylogenetic analysis suggests that the G duplication variants emerged within the last decade, with the 111-nt first appearing in Japan, Croatia, and China, and the 180-nt duplication first reported in Spain [104,106,107,108,109]. These strains have rapidly become the dominant circulating strain in multiple geographic regions. Analogous duplication events in the attachment G glycoprotein have also been described in RSV, where they are similarly associated with rapid lineage expansion, supporting a recurring evolutionary strategy among *Pneumoviridae* [110,111]. Despite their rapid expansion and apparent selective advantage, functional studies defining the G duplication’s viral fitness remain limited [105]. This widespread diversity, particularly in aMPV, is thought to be driven by the introduction of vaccination programs, and it can impact the efficacy of regional live-vaccine vaccination programs [97,101].

### 3.9. L Protein

The L gene is the largest and encodes the viral RNA-dependent RNA polymerase. It contains the canonical polymerase domain and enzymatic machinery for genome replication and performs nucleotide polymerization, cap formation, polyadenylation, and mRNA synthesis [57,58,112]. It associates with P and encapsidates RNA to perform replication of the negative-strand genome [57,58]. The sequence and domain structure of L are strongly conserved across both hMPV and aMPV, with aMPV/C isolates sharing 80% amino acid identity with hMPV and 64% amino acid identity with other strains of aMPV [44,52,113]. This level of conservation shows the importance of key enzymatic mechanics and structural constraints necessary for polymerase function and supports the use of L sequences for robust phylogenetic placement and for inference of deep evolutionary comparisons between aMPV and hMPV strains [113,114].

### 3.10. Role of Different Proteins in Host Cell Entry and Immune Evasion

**Figure 3:**
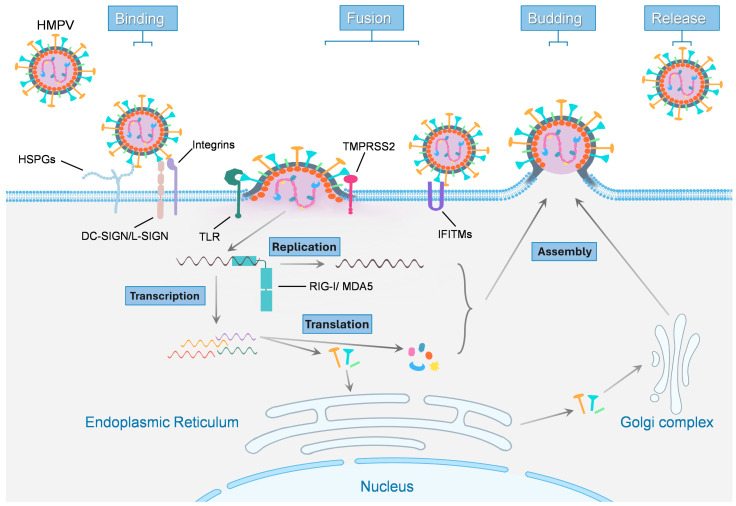
Metapneumovirus cell entry. Replication is initiated through attachment to host cell surface molecules, including heparan sulfate proteoglycans (HSPGs), C-type lectins (DC-SIGN and L-SIGN), and RGD-binding integrins. The viral G protein mediates initial attachment by engaging negatively charged HSPGs, while the F protein enhances attachment and triggers membrane fusion through conformational rearrangements and interactions with integrins such as αVβ1, αVβ5, and αVβ8. Following attachment, hMPV can enter cells either by direct fusion at the plasma membrane (as seen above) or by endocytosis with subsequent fusion within endosomal compartments, depending on the cell type and entry pathway. In monocyte-derived dendritic cells, macropinocytosis is the primary route of entry for hMPV, it can additionally exploit DC-SIGN-mediated endocytosis as an alternative pathway for entry and infection. Fusion releases the viral genome into the cytoplasm, where it serves as a template for transcription and replication by the viral polymerase complex. Newly synthesized genomes associate with nucleocapsid proteins (N, P, L, and M2) and the matrix protein (M), while the F, G, and SH glycoproteins are processed through the endoplasmic reticulum and Golgi apparatus prior to assembly and budding. Host antiviral restriction factors, including IFITM proteins and cytosolic RNA sensors such as RIG-I and MDA5, act at early stages to limit viral entry and replication, while other host factors like toll-like receptors (TLR) and transmembrane protease serine 2 (TMPRSS2) have been shown to further facilitate hMPV infection and evasion of the host immune system. Figure reproduced from [76]. Licensed under the Creative Commons Attribution License (CC BY); high-resolution image generously provided by the authors.

Metapneumoviruses enter host airway cells via fusion at the plasma membrane or late endosome following receptor engagement. Replication of metapneumoviruses is initiated by viral attachment and entry, processes mediated by the surface glycoproteins G and F. These proteins facilitate binding to host cell receptors and trigger fusion between the viral envelope and the host cell membrane, resulting in the delivery of the negative-sense RNA genome into the cell’s cytoplasm (Figure 3). The viral attachment and entry are key determinants of cellular tropism and significantly influence downstream replication efficiency and host responses.

Early and recent work has identified heparan sulfate proteoglycans (HSPGs) as key attachment factors for hMPV on many cell types, with the viral F protein sufficient for binding [115,116,117]. Enzymatic removal or competitive inhibition of heparan sulfate reduces viral attachment and infection in cell culture and airway tissues [115]. Beyond HSPGs, the F protein contains an RGD motif and interacts with RGD-binding integrins, facilitating closer virion contact with the cell for entry [117,118]. The F protein triggers membrane fusion after binding to integrin or lectin host receptors, which is dependent on the strain [76,77]. Protease-sensitive proteinaceous receptors also appear to contribute to infection, with some indications that the presence of a trypsin-sensitive cell surface protein required for optimal infection by hMPV exists in certain cell types [115,118]. These findings support a model in which metapneumoviruses utilize multiple, partially redundant, and cell-type-specific attachment and entry cofactors to establish a productive infection.

Recent glycan-binding profiling has expanded this view by demonstrating hMPV binds to a diverse set of glycans beyond classical heparan sulfate motifs. This broad glycan-binding capacity likely contributes to infection of multiple epithelial subtypes and provides flexibility in viral attachment strategies [119,120]. Studies show that hMPV entry can occur via direct fusion at the plasma membrane or following endocytosis and fusion within endosomal compartments, with the entry route being cell-type dependent [118,121].

Following entry, the viral ribonucleocapsid is released into the cytoplasm, where transcription and replication occur. Transcription initiates at the 3′ promoter and proceeds sequentially through the genome, producing capped, polyadenylated mRNAs in a gradient of decreasing abundance [122,123]. Each gene encodes a single open reading frame (ORF) except M, which contains 2 ORFs. The 3′ leader and 5′ trailer regions serve as promoters for replication [26,52]. The polymerase complex (L-P-N) synthesizes full-length antigenomic RNA, which in turn replicates into progeny genomes. Newly synthesized RNPs complex with M at the cell membrane through interactions between M and the RNP to form virions, which bud off as enveloped particles with embedded F, G, and SH glycoproteins [52]. In parallel, the envelope glycoproteins F, G, and SH are synthesized and processed through the endoplasmic reticulum and Golgi apparatus. Assembly of viral components occurs in the plasma membrane, culminating in the release of mature virions through budding.

For each of these processes, all viral proteins can modulate the host cell. During hMPV infection, the host IFN response is downregulated by the G and SH proteins that collaborate to block IFN induction and signaling. hMPV also modulates cytokine networks as SH and G jointly impair JAK/STAT and RIG-I pathways [96,124]. Though the details in avian species are less characterized, aMPV likely employs parallel strategies in avian cells. While further studies need to be completed, especially for each subtype, the aMPV/C G protein has been shown to play a role in virus–host immune interactions [52]. In all cases, the matrix and polymerase proteins are central for viral assembly and replication, with envelope proteins dictating cell tropism and immune recognition.

## 4. Host Range and Tropism

### 4.1. Host Range and Tropism of aMPV

Avian metapneumovirus causes a highly contagious upper respiratory infection in a wide range of domestic and wild birds, with a marked tropism for ciliated columnar epithelial cells of the nasal cavity, sinuses, trachea, and, to some extent, the oviduct epithelium of laying birds [125,126]. Viral antigen and genome are consistently detected in the apical region of the ciliated epithelial cells, and productive replication is associated with loss of cilia and degeneration of the epithelial layer [125,126,127]. Tissue and strain differences influence viral tropism, as some of the aMPV strains replicate more efficiently in oviduct tissues while others replicate better in tracheal epithelium, indicating both tissue-specific and strain-specific tropism [125,126,127,128]. Gross lesions are localized primarily in the upper respiratory tract and periocular or perinasal tissues and often include mucosal hyperemia, conjunctival chemosis, and periorbital or facial swelling [125,129,130]. This facial swelling, most notable around the eyes and beak in infected chickens, is how the virus earned its original name of swollen head syndrome. In severe outbreaks, particularly when secondary bacterial infections such as *Escherichia coli*, *Mycoplasma gallisepticum*, *Mycoplasma synoviae*, *Bordetella avium*, and *Ornithobacterium rhinotracheale* follow primary viral infection, fibrinous or caseous subcutaneous exudate and osteomyelitis in head bones and upper respiratory tissues may be seen [128,129,131,132,133,134,135]. Reproductive lesions in laying birds can be subtle grossly but include oviduct inflammation and decreased egg quality and production. Visible caseous material can occasionally be seen within the oviduct [126,131].

Microscopically, the earliest and most consistent lesion is ciliostasis with progressive loss or fragmentation of cilia on respiratory epithelial cells, accompanied by epithelial cell degradation and necrosis [125]. Acute tracheitis and rhinitis are characterized by epithelial desquamation, mucosal erosion, submucosal edema, and infiltration with heterophils and mononuclear inflammatory cells [125,131]. Viral antigens are frequently detected by immunohistochemistry along the apical membrane and ciliary border of infected epithelial cells, which is consistent with the strong tropism of aMPV for ciliated epithelium. Persistent antigen expression corresponds with ongoing epithelial injury and lesion severity [126]. Secondary lesions in the lower respiratory tract, such as bronchi, parabronchi, and air sacs, are variable and are more commonly reported when co-infections occur [130]. Severe coughing from lower respiratory tract infections can, however, lead to prolapsed uteruses in breeding turkeys [136]. Reproductive tract histopathology includes epithelial necrosis in the oviducts, inflammatory infiltrates in the mucosa and submucosa, and occasional glandular atrophy changes that impact eggshell quality and drop in egg production [9,126,131].

### 4.2. Immune Response

The aMPV infection elicits a mixed innate and adaptive immune response in birds. Innate responses include induction of interferons and other pro-inflammatory mediators in the respiratory mucosa, while adaptive responses involve local mucosal and systemic antibody production [8,125,136,137]. Organ-culture studies have shown an upregulation in infected tissues of type I and type III interferons, like IFN-α and IFN-λ, along with inducible nitric oxide synthase, which corresponds with decreasing viral replication and the onset of epithelial repair [125,126,137]. Cytokine profiling in infected turkeys has shown that increased IFN-γ production by T lymphocytes in the spleen and upper respiratory tract, while regulatory cytokines like IL-6 and IL-18 are significantly upregulated in turbinate and trachea tissues, conferring both antiviral and immunomodulatory pathways, are upregulated after early infection [138,139]. The balance between CD4^+^ and CD8^+^ T-cell responses appears to depend on prior antigen exposure based on maternal antibodies (MDA). In MDA-naïve turkeys, vaccination or infection stimulates CD8^+^ infiltration in the respiratory mucosa, whereas in MDA-positive birds, the response skews heavily towards CD4^+^ T cells [137,139,140].

Both aMPV and hMPV induce measurable mucosal antibody responses following natural infection or vaccination, including the expansion of IgA^+^ B cells and the detection of virus-specific IgA in respiratory secretions [140,141,142]. In aMPV-infected or vaccinated birds, IgA is detectable in mucosal secretions, such as tracheal wash and nasal fluid [138,140]. MDA also plays a role in the magnitude and quality of this response, as birds with high MDA levels often show reduced induction of mucosal IgA and systemic IgG following vaccination, likely due to early viral neutralization limiting antigenic stimulation [140,142,143,144]. However, localized IgA and systemic IgG do not fully predict protection, as passively transferred antibodies via serum or mucosal surfaces do not reliably provide complete protection to prevent clinical disease, especially in turkeys [142,145]. Clinical reinfections are often associated with reduced disease severity rather than prevention of infection, suggesting that IgA contributes to modulation of clinical outcome but does not reliably prevent viral entry or early replication in the respiratory epithelium [145]. This implies that the humoral immune reaction is not enough on its own and that other immune mechanisms, like localized mucosal immune responses and cell-mediated immunity, are also essential for effective aMPV and hMPV protection [8,142,146].

### 4.3. Clinical Signs

Clinical signs include nasal discharge, coughing, sneezing, conjunctivitis, opisthotonus, swollen sinuses, and ruffled feathers from lack of grooming [131,135]. The species of bird infected does have some impact on the clinical signs, as turkeys often show more overt and severe signs than chickens, and have more pronounced respiratory distress and higher morbidity outcomes [130,131]. Metapneumoviruses have similar clinical signs to pneumovirus infection; they lack the NS1/NS2 genes found in related pneumoviruses like RSV, which may factor into the differences in host immune response [2].

### 4.4. Transmission

The aMPV is highly contagious and transmits efficiently by respiratory secretions, direct contact between birds, fecal-oral exposure, and fomites contaminated with nasal or ocular discharge, leading to rapid spread in a flock once the virus is introduced [146,147]. Mechanical vectors such as contaminated personnel, equipment, and possibly wild birds are major factors for flock-to-flock transmission and are often the leading culprits in new outbreaks [12,148,149,150]. Clinical disease develops rapidly after flock exposure, and in some of the most extreme cases, a flock of 10,000 turkeys is completely infected in as little as 24 h after exposure [150]. Anecdotally, poultry growers have been known to say that “if your neighbor has aMPV, you already have it”, which speaks to the observations in the field on just how fast aMPV can spread in some areas.

Outbreaks are characterized by high morbidity and low to moderate mortality. Deaths that do occur during outbreaks are most often due to dehydration, starvation, or opportunistic secondary bacterial or viral infections such as airsacculitis, pericarditis, Newcastle disease, cholera, *Bordetella avium*, *Mycoplasma* spp., and *Escherichia coli.* Severe aMPV infections predispose infected animals to secondary infections, which have major impacts on survival rates and overall flock health, often leading to increased condemnation rates at slaughter [126,129,135].

Infection can occur year-round; however, the geographic location and climate can play a role in seasonal infection patterns. In general, outbreaks are often associated with the fall and spring in line with seasonal migratory patterns of wild birds or coinfection with other seasonal disease outbreaks like influenza or airsacculitis [148,151,152]. Age plays a major role in susceptibility, as young poults and chicks typically develop more pronounced clinical disease with severe epithelial lesions, whereas older birds and adults often exhibit milder symptoms or subclinical infections. Maternal antibodies provide variable early protection but do not always confer complete protection against infection, and the protection is strain-dependent [146,153]. These factors can complicate control strategies in multi-age or high-throughput production systems.

Both hMPV and aMPV exhibit strict host specificity, reflecting adaptation to humans versus avian hosts. While it is not known to naturally infect birds or other mammals, experimental inoculation of turkeys with hMPV has shown they can be infected under laboratory conditions, and isolated spillover events between humans and non-human primates (NHP) have been reported [154,155,156,157,158]. Conversely, aMPV infections from birds have not been reported to cross over to humans or other mammals. These host barriers are likely due to differences in receptor usage, body temperature, pH preference, and innate immune recognition. Both viruses target the respiratory epithelium, but differences exist in their viral entry pathways. Host immune environments also differ; hMPV glycoproteins are tuned to evade human innate sensors and intermediate signaling molecules like STAT1 and RIG-I, whereas aMPV, while less studied, is presumed to counter avian innate responses [95,96]. A summary of comparative immunological characteristics between aMPV and hMPV can be found in Table 2.

### 4.5. Host Range and Tropism of hMPV: Humans

Human metapneumovirus infects humans of all ages and represents a significant public health burden across all age groups, particularly in young children, elderly adults, and immunocompromised individuals [5,159]. It causes upper respiratory symptoms, including runny nose, persistent cough, and sore throat, and frequently leads to bronchiolitis or pneumonia in infants and young children [5,6,160,161,162]. Nearly 100% of children have hMPV antibodies by age five, but immunity is short-lived, which means that recurring infections throughout life are common [5]. In the U.S., hospitalization rates of 1 per 1000 children and 3 per 1000 infants under 6 months old, with over 1 million outpatient and emergency department visits, especially during the peak of the infection season between winter and spring [163,164,165]. It is second only to RSV as a cause of severe pediatric lower respiratory infection [166,167]. In hospitalized pediatric populations, hMPV infection is associated with clinically significant morbidity, with over 263,000 cases yearly: intensive care admission makes up 18%, mechanical ventilation is needed in 6%, 40% of children hospitalized had underlying high-risk conditions, and median hospital stays are around 2.8 days on average [164]. In healthy adults, hMPV usually causes mild cold-like symptoms, but in older or immunocompromised adults, the burden can be similar to children [168]. Approximately 122,000 adults aged 65 years or older were hospitalized in 2019 in the U.S. due to hMPV infection [159,169].

The economic burden is also high, with projections for hMPV-driven hospitalizations in the U.S. alone costing $277 million per year [170]. The true impact of hMPV on a global scale for all vulnerable populations is currently unknown, as global tracking of hMPV is severely lacking. Surveillance studies have repeatedly reported a lack of publicly available information on hMPV spread on a global scale, with one study finding that only 26 countries reported suitable surveillance data that could be used for global surveillance work [163,169,171]. To complicate global tracking further, RSV and hMPV cocirculate seasonally in many temperate regions, often with overlapping peaks in late winter and spring [163,165,172]. Both viruses target similar epithelial cell populations in the lower respiratory tracts and elicit partially overlapping innate immune responses, which may predispose to more severe clinical disease when sequential or concurrent infections occur [165]. Clinical surveillance studies have described increased hospitalization risks when one virus follows the other within a short interval in the same region, suggesting transient immune modulation that reduces effective antiviral control [163,165]. This creates diagnostic challenges as both RSV and hMPV present with comparable clinical symptoms in similar vulnerable populations, increasing the likelihood of misattribution and underestimation of dual infections when molecular testing is lacking [18,172]. Co-infections of RSV and hMPV, while reportedly infrequent, have been associated with prolonged illness, higher rates of hypoxia, and increased need for respiratory support, especially in pediatric patients [173,174,175,176]. Simultaneous or overlapping circulation of RSV and hMPV amplifies seasonal strain on healthcare resources, particularly in pediatric wards, and complicates forecasting models for respiratory disease burden [163,165,172,176]. With the potentially successful development of RSV vaccines and monoclonal antibodies, additional considerations for whether selective reduction of RSV incidence will shift competitive viral dynamics and alter the timing and magnitude of hMPV outbreaks should be taken into consideration by health care providers [172,177,178,179,180].

Within the human respiratory tract, hMPV demonstrates a broad epithelial tropism. In vitro and in vivo studies show efficient infection of ciliated airway epithelial cells in the upper airway and of bronchiolar and alveolar epithelial cells in the lower respiratory tract [181,182,183]. Alveolar macrophages and dendritic cells are frequently implicated as infected or activated cell populations, contributing to downstream inflammatory responses and cytopathic changes that disrupt Toll-Like receptor (TLR) dependent signaling and Type I IFN production [184,185,186]. In mice and macaque models, hMPV infection of alveolar macrophages and dendritic cells does not always result in high-titer replication but can potentially remodel local cytokine and chemokine milieus, shaping leukocyte recruitment and disease severity [181,187,188,189]. Organoid models have confirmed that hMPV can replicate in differentiated respiratory epithelia and that replication efficiency and innate immune responses vary with cell type and their differentiation state [190]. Comparisons between aMPV and hMPV tissue tropisms can be found summarized in Table 3.

## 5. Diagnostics

Quantitative reverse-transcriptase PCR (qRT-PCR) remains the diagnostic gold standard for both hMPV and aMPV [191,192,193]. For hMPV, in particular, it is generally part of a larger multiplex panel for syndromic diagnosis. In humans, nasopharyngeal swabs and aspirates are most frequently used due to ease of collection and high viral load during early respiratory infection, whereas sputum, bronchoalveolar lavage (BAL), and endotracheal aspirates may yield higher sensitivity in severe lower respiratory disease or in immunocompromised patients [194,195,196,197,198]. In poultry, tracheal and/or oropharyngeal swabs collected during acute infection are optimal, although turbinate or tracheal tissues may also be tested post-mortem [125]. The G protein is highly variable and prone to deletions or substitutions, which can cause primer mismatch and reduced sensitivity in G-targets assays [100,199,200]. Instead, validated, commercially available RT-PCR assays generally target more conserved proteins, such as the N, M, or F, which provide high sensitivity across strains [191,201,202]. Multiplex RT-qPCR panels are widely available for clinical use in single-plex and multiplex assays for both hMPV and aMPV [203,204,205,206].

Rapid antigen detection tests and direct immunofluorescent assays provide faster results than RT-PCR; however, they usually have lower sensitivity, especially for hMPV in adults with low viral loads, and are rarely used in clinical practice [198,207]. Serological assays like Enzyme-linked Immunosorbent Assays (ELISAs) or viral neutralization are primarily used as epidemiological tools because acute diagnosis requires paired sera to demonstrate seroconversion [208].

Viral isolation, through LLC-MK2 cells for hMPV and Vero cells for aMPV, is a labor-intensive process that has a slow turnaround time and is rarely, if ever, used for routine testing settings [184,209]. Its early usage in surveillance might have been a contributing factor as to why hMPV remained undiscovered for so long, as hMPV has a relatively late showing of CPE compared to many viruses. In aMPV, virus isolation samples tend to need to be collected at certain times for positive results, and CPE was rarely seen even after multiple passages, with early researchers noting it could take up to two months for primary isolation of the virus [7,209,210,211,212]. It is now reserved mainly for research or phenotypic characterization of aMPV and hMPV [18,192,213].

Several recent advances have enhanced diagnostic performance into new potential platforms for surveillance and research. Droplet digital RT-PCR (ddPCR) provides absolute quantification and superior performance in low-titer samples, with potential utility for viral load monitoring and multi-plex assays to detect other respiratory viruses [214,215,216]. Amplicon-based whole-genome sequencing (WGS), adapted from SARS-CoV-2 surveillance protocols, enables the near-complete genome sequencing of hMPV and aMPV directly from clinical or field samples without the need to culture them [29,217,218]. This approach facilitates the potential for phylogenetic analysis, outbreak tracing, and identification of mutations affecting diagnostic primers or antigenicity. Integrated point-of-care molecular platforms are increasingly incorporating hMPV detection, providing rapid and sensitive results in decentralized settings [217,219].

It is important to note that genetic variability influences overall assay performance. The major groups and sublineages of hMPV, in addition to the aMPV subtypes, have made a universal test difficult for either virus. An example of this struggle is best seen in the aMPV/C outbreak in North America, which began in 1996 [220]. Early diagnostics and treatments were ineffective at identifying or treating the outbreak, as the only known tests at the time tested for aMPV/A or aMPV/B. Several laboratories showed that aMPV/C was distinctly different in sequence, and that it was so unique that current tests and treatments proved ineffective and required the development of new diagnostic tests and vaccines [209,220,221,222,223,224,225].

### Sequence-Based Genotyping and Molecular Epidemiology of hMPV

Sequencing data, whether from targeted gene amplification or complete viral genomes, has enabled detailed genotyping and better molecular epidemiology analysis of hMPV. Classical classification relies on partial or full-length F and G sequences. The F gene, being moderately conserved, is used for robust lineage assignment, while the highly variable G gene provides details of sublineages and identification of lineage-specific nucleotide duplications, such as the 111-nt or 180-nt G-gene duplications [106,107,226]. 

Phylogenetic inference using maximum-likelihood or neighbor-joining methods allows researchers to place novel sequences in the context of global reference datasets and track temporal shifts in circulating genotypes [4,226]. High-resolution WGS data sets also enable the detection of co-circulating sublineages and the identification of mutations that may affect antigenicity or diagnostic assay performance [29]. 

Bioinformatic tools adapted for hMPV include Nextstrain for real-time phylogenetic visualization and clade assignment. Currently available programs like MAFFT for sequence alignment, IQ-TREE and MEGA for phylogenetic reconstruction, and BEAST for Bayesian phylogenetic analysis of temporal and population dynamics have been integrated into many hMPV projects to provide a comprehensive framework for understanding viral evolution and molecular epidemiology in human populations [4,226,227,228,229,230,231,232].

## 6. Vaccine Strategies

### 6.1. Vaccine Strategies for hMPV

No licensed vaccines currently exist for hMPV despite the significant global burden of hMPV-associated respiratory infections [169,233]. Vaccine development has been hampered by several challenges, including antigenic diversity of circulating genotypes, limited understanding of correlates for protection, and the risk of enhanced respiratory disease observed historically with formalin-inactivated vaccines against closely related paramyxoviruses like RSV. Notably, recent advancements in RSV vaccine development have reshaped hMPV vaccine design strategies [234,235,236,237,238]. The F protein of hMPV and RSV F are class I fusion glycoproteins that undergo a metastable transition from a pre-fusion to post-fusion conformation during viral entry, as previously shown in Figure 1. Retrospective analyses of the RSV formalin-inactivated vaccine from the 1960s revealed that immune responses were largely directed against the post-fusion F confirmation, resulting in poorly neutralizing antibodies and enhanced disease upon natural infection [234]. These findings ultimately drove the development of modern-day structure-guided approaches that stabilize the pre-fusion F confirmation, which is now a cornerstone of successful RSV vaccines [237,238]. hMPV differs from RSV in that it has the potential neutralizing epitopes in both pre- and post-fusion F confirmations; however, in general, these advances in RSV research have been directly translatable to hMPV [239]. Analogous pre-fusion F stabilization strategies are being applied to improve antigenicity and immunogenicity across multiple vaccine platforms, including subunit, mRNA, and live attenuated candidates.

Subunit vaccines utilize purified viral components to elicit protective immunity without introducing the whole virus. The hMPV F glycoprotein is the principal target of neutralizing antibodies and a favored vaccine antigen target because it is highly conserved and mediates membrane fusion [63,240,241,242]. Using a similar approach that has led to major advances in the RSV vaccine, subunit vaccines utilize recombinant prefusion-stabilized F proteins and virus-like particles (VLPs) to produce prefusion-stabilized F constructs that elicit potent neutralizing responses in animal models and preliminary translational studies [235,240,242,243,244,245]. These engineered pre-F immunogens restore and present neutralization-sensitive epitopes that are poorly displayed in post-fusion conformations [236,243]. Subunit vaccines minimize the risk of vaccine-associated disease by utilizing only defined singular viral proteins; they tend to have reduced immunogenicity compared to live, to whole organism vaccines, and they require an adjuvant or carrier to be effective [246,247].

The successful advent of nucleoside-modified mRNA vaccine platforms since the SARS-CoV-2 pandemic has accelerated the development of vaccines for many respiratory viruses, like RSV and hMPV [180,248]. mRNA-based vaccines offer several conceptual advantages as a vaccine platform: intracellular expression of the antigen can mimic native viral antigen processing, potential induction of both humoral and cellular immunity, scalable manufacturing, and the ability to update quickly to match emerging strains [248]. However, several challenges remain as the immunodominance of pre-F epitopes needs long-term safety evaluation and dose optimization for infants, and the elderly must be considered to avoid risks of vaccine-associated enhanced respiratory disease, as seen in previous RSV vaccine attempts [234,248,249]. A current leading candidate, mRNA-1653, encodes the full-length F protein of hMPV and human parainfluenza virus 3 (HPIV3) in a single lipid nanoparticle formulation [249]. It is currently in clinical trials, where phase 1 studied healthy adults ages 18–49, and was well-tolerated across multiple dosage levels. A single dose was found to boost neutralizing antibody titers against hMPV subgroups by a geometric mean of 6-fold. Titers remained above baseline for 13 months post-vaccination, demonstrating encouraging long-lasting protection [249].

Historically, live attenuated vaccines have demonstrated promising immunogenicity and protective efficacy in animal models, including hamsters, mice, and non-human primates, while maintaining acceptable attenuation profiles [98,99,250,251]. They have generally been developed using reverse-genetics systems to delete non-essential accessory genes like SH or G and introduce attenuating mutations [98,99,252,253]. These manipulations aim to reduce virulence while preserving immunogenicity. While most efforts of a live-attenuated vaccine have shifted to different vaccine platforms, one prominent current vaccine candidate has combined an hMPV SH backbone with an exogenous F gene from RSV to produce a bivalently mucosal vaccine that protects against both viruses in mice [254]. The potential to target multiple co-circulating respiratory viruses in one vaccine could offer broad coverage and significantly reduce the health burden caused by both hMPV and RSV, and lower the risk of an increase in the circulation of one virus when removing another [163,165,172,254]. Live attenuated vaccines induce robust, long-lived mucosal and systemic immunity that elicit strong T-cell responses and mimic natural infection routes more closely than non-replicating platforms, potentially offering a stronger immune response [254,255,256]. Efforts to develop potential RSV vaccines have shown that live-attenuated vaccines are easily administered intranasally in humans compared to other vaccine platforms, which is considered a preferred route of inoculation in pediatric populations compared to intramuscular injection, as it is non-invasive and can facilitate mass vaccination programs more readily [254,257,258]. The main challenge for hMPV live-attenuated vaccines is the same challenge that any live-attenuated vaccine encounters, e.g., proper attenuation to provide protection while maintaining genetic stability to avoid reversion and overcoming regulatory hurdles for safety in infants and immunocompromised individuals while eliciting long-lasting protection.

### 6.2. Vaccine Strategies for aMPV

In contrast to hMPV, vaccines for all circulating subtypes of aMPV are widely available and are routinely used in the poultry industry [138,259,260]. There are two main types of aMPV vaccines currently used in the industry: live-attenuated and inactivated vaccines. Other vaccine platforms like mRNA vaccines do not currently exist; recent efforts in alternative platforms for hMPV may drive the development of similar new vaccine types for aMPV as well. A full comparison of vaccine strategies between aMPV and hMPV can be found in Table 4.

Live attenuated vaccines, generated by serial passage or cold adaptation of field isolates, are commonly deployed via aerosol, coarse spray, in drinking water, or oculonasal routes to induce rapid mucosal immunity and cell-mediated response at the respiratory mucosa. These vaccines are currently the most commonly used in preventing aMPV infections and confer a prompt reduction in clinical signs and transmission in flocks [138,260]. They are advantageous to hatchery operations with day-old administration and are best suited for short-turnover poultry systems such as broiler or meat-turkey production, since they typically require a single dose to achieve a useful reduction in disease expression [261]. Live vaccines have shown cross-protection for aMPV/A and aMPV/B subtypes and can be co-administered with other vaccines for other diseases with no loss of protection [262,263]. They can circulate transiently through a flock and can be shed by vaccinated birds, which can complicate molecular surveillance if the vaccine strains are not distinguishable from field viruses [264]. This can also lead to mild disease seen in otherwise vaccinated flocks and can, in some cases, even revert to full virulence after several passages in a flock, causing an outbreak [265,266,267,268,269]. Reversion to virulence is of particular concern if imported live vaccines made using foreign strains are used, as it can introduce new types of virulent viruses to a new area [268,269]. This leads to complications in the potential usage of foreign live attenuated vaccines, and oftentimes, foreign vaccines are strictly regulated and only used if no other options are readily available.

Inactivated oil-adjuvanted vaccines are primarily used in countries that do not allow live vaccines and in breeders and layers to elicit high systemic neutralizing antibody titers that are transferred to progeny via the egg yolk by MDA [140,270]. They provide predictable systemic humoral responses without the risk of replication or shedding, but they are less effective at preventing upper respiratory replication and shedding compared to live vaccines [140,142,143]. They usually require two doses to reach peak efficacy, which in turn requires labor-intensive injection schedules that are often impractical for large-scale broiler operations [143,144].

Contemporary vaccination programs for aMPV must account for the diversity in circulating field strains. Commercial flocks have documented natural infection with field strains despite rigorous vaccination programs, indicating that conventional vaccines may not always prevent infection or shed virus when antigenic match to the circulating field strain is suboptimal [271,272,273]. Recent advances in aMPV vaccine development have focused on enhancing cross-protective immunity across subtypes A and B. Newer live-attenuated vaccine strains continue to show heterologous protection in controlled experimental systems [138,274,275]. While cross-protection has been demonstrated repeatedly under controlled laboratory conditions, translation to commercial production environments remains a gap in the literature. Current commercial vaccines are still largely evaluated under controlled settings, and field data showing efficacy against wild-type viruses from a diversity of regions are limited. Ongoing molecular surveillance and subtype characterization are critical inputs for updating vaccine strain composition and predicting cross-protective potential in real-world settings. Increased diversity in viral test strains against these cross-protective vaccines would also confirm the protective nature of these vaccines and lead to better vaccination programs in the field.

The timing of vaccine administration and the interplay with MDA are a major determinant in successful vaccine programs in commercial systems [139]. In turkeys, high MDA levels from inactive vaccines in breeders protect neonates from early clinical disease but can substantially reduce the success of live-attenuated vaccines and blunt mucosal IgA induction when live vaccines are given at hatch or to one-day-old poults [144,145]. Unvaccinated birds with MDA can still become infected, indicating that MDA protection by itself is incapable of preventing infection [140]. MDA positively correlates with lower success with live attenuated vaccines, as maternally immune birds have shown more rapid clearance of maternal antibodies but less robust immunoglobulin induction, suggesting that MDA has a neutralizing effect on vaccination timing [145]. Vaccination programs that utilize inactivated boosters to elevate maternal titers while scheduling live vaccination of progeny at a point when MDA has waned sufficiently to permit vaccine virus replication or use heterologous live strains to mitigate interference and maximize flock protection [143]. The balance between providing immediate passive protection and enabling effective active immunization in progeny requires serological monitoring and locally adapted schedules within each operation rather than a single universal timetable because the window of MDA-mediated interference and the overall efficacy of vaccination programs vary with breeder vaccine history, maternal titer magnitude, production cycle length, species of bird, and seroprevalence of regional aMPV strains [139].

Optimization of aMPV vaccination schedules in poultry has increasingly focused on measurable immunological markers that correlate with protective immunity rather than solely on age or MDA decline. Traditional serological measures, such as serum IgG titers alone, are insufficient predictors of protection. Mucosal and cellular responses, particularly IgA expression in respiratory tissues and activation of T-cell subsets, are more closely associated with functional immunity following vaccination or infection in aMPV. Transcriptomic and cellular markers in the respiratory mucosa have also proven to be informative in evaluating vaccine-induced responses. Following subtype B vaccination, studies in chickens indicate upregulation of mucosal immunoglobulin transcripts such as IgA and IgY, and pro-inflammatory cytokines like IL-1β, IL-6, and IL-18 after challenge [138,139,276]. These may serve as better correlates of active mucosal engagement and post-vaccination responses. Cellular immunity, quantified by CD8-α and CD8-β transcriptional responses in respiratory tissues, may reflect T-cell activity that contributes to viral clearance and reduction of shedding, signaling more effective vaccine-mediated immune priming [138,139].

Emerging methods assessing functional activity of tracheal explants as a surrogate for clinical protection offer an alternative measure of vaccine efficacy beyond conventional serology. This approach has shown promise in evaluating the protective impact of vaccination on respiratory epithelial function in birds from 17 days to 16 weeks old and may serve as an additional biomarker in field strains [276,277].

## 7. Conclusions

### Conclusions and Recommendations

Metapneumoviruses occupy a unique position within respiratory virology, distinguished by their shared genomic organization and core replicative functions, yet still having a remarkable diversity that makes the virus species-specific. The parallels between hMPV and aMPV provide a framework for understanding how metapneumoviruses replicate, are transmitted, and persist across diverse host species, while differences in receptor usage, immune modulation strategies, and glycoprotein variability reflect the selective pressures shaping their evolution over time. In avian systems, aMPV continues to pose a major challenge for poultry production globally, particularly in regions where subtype diversity and dynamic antigenic profiles undermine vaccine effectiveness and complicate long-term control efforts. In contrast, hMPV remains a leading cause of respiratory disease in both young and old humans, yet no licensed vaccines or targeted antiviral therapeutics are currently available, underscoring a persistent translational gap despite decades of virological and immunological research.

Comparative analysis underscores how host immune landscapes and intervention strategies intersect with viral genetic diversity to shape disease outcomes and control success. Extensive molecular and genomic data now exist for both viruses, yet the functional consequences of sequence variation, particularly within highly variable regions involved in immune recognition and modulation, remain incompletely understood. This disconnect between genomic characterization and phenotypic interpretation limits the ability to predict vaccine performance and potential immune escape in both human and avian systems. Addressing these limitations will require coordinated efforts that integrate molecular surveillance with functional genomics and immunological profiling to scale aMPV and hMPV tracking to match the same global database size that other respiratory diseases like influenza and SARS-CoV-2 already have in place. Platforms, such as Nextstrain, have already transformed tracking of hMPV lineage dynamics and could be leveraged to monitor aMPV to enable a better understanding of antigenic drift and regional transmission patterns in poultry systems. Increased surveillance for molecular testing to positively identify hMPV and aMPV cases, combined with further sequencing of isolates, would strengthen the overall understanding of circulating strains in both human and avian populations. 

Looking forward, cross-disciplinary and comparative approaches offer a clear path toward advancing the field beyond descriptive characterization. Defining robust molecular correlates of protection, functional mapping of genotype–phenotype relationships, incorporating real-time genomic data into diagnostic and vaccine design, and tracking subtype emergence represent critical priorities for both hMPV and aMPV research. By leveraging shared features and host-specific differences across metapneumoviruses, future studies can develop predictive, mechanism-driven strategies that improve disease control in poultry systems while informing vaccine and therapeutic development for human populations. Collectively, such efforts have the potential to reduce disease burden across species and establish a more unified framework for understanding and mitigating metapneumovirus-associated respiratory diseases.

## Figures and Tables

**Table 1:** Genome Comparison of hMPV and aMPV.

Gene/Protein	Core Function	aMPV Characteristics	hMPV Characteristics	Approximate Amino Acid Identity	Key Comparative Notes
**Genome**	Negative-sense RNA genome	~13 kb; conserved gene order	~13 kb; conserved gene order	N/A	Nearly identical genome length and architecture across species, supporting a shared evolutionary origin
**N (Nucleoprotein)**	RNA encapsidation; RNP formation	Highly conserved; strong RNA binding; scaffolds polymerase complex	Highly conserved; identical functional role in RNP	~70–99%	Some aMPV/C and hMPV strains approach near identity, indicating exceptional conservation
**P (Phosphoprotein)**	Polymerase cofactor; N chaperone; inclusion body formation	Moderately conserved; coordinates N–L interactions; induces cellular extensions	Similar multifunctional role in replication compartment organization	~67–68% (aMPV/C vs. hMPV); lower vs. other aMPV	Conservation sufficient for shared mechanisms, but divergence may influence host-specific replication dynamics
**M (Matrix protein)**	Virion assembly and budding	Structural role conserved; immunomodulatory effects are less defined in avian hosts	Structural role plus documented immune activation via extracellular M	~87–88% (aMPV/C vs. hMPV)	High conservation reflects strong structural constraints across hosts
**F (Fusion protein)**	Membrane fusion; viral entry	Subtype-specific integrin motifs (RDD in aMPV/B; RSD in aMPV/C); pH-independent fusion	Contains RGD motif, requires low pH for fusion	~56–61% (aMPV/C vs. hMPV)	Conserved fusogenic core with divergent receptor usage and entry requirements
**M2-1**	Transcription elongation; antitermination	Essential for transcription; zinc-binding CCCH motif	Same essential transcriptional role	~52–70%	Functional conservation despite moderate sequence divergence
**M2-2**	Regulation of transcription vs. replication; immune evasion	Inhibits host transcription and IFN signaling	Similar regulatory role	<20%	Marked divergence suggests host-specific immune modulation strategies
**SH (Small** **hydrophobic** **protein)**	Immune modulation; viroporin activity	Highly variable; extreme divergence among subtypes	Variable but more conserved than aMPV	~14–31%	Likely conserved archetype with highly divergent host-interaction motifs
**G (Attachment** **glycoprotein)**	Attachment: immune evasion	Extremely diverse; strong subtype divergence; vaccine-driven variability	Highly variable; frequent duplication events (111-nt, 180-nt)	~20–30%	Most divergent protein; major contributor to antigenic and evolutionary differences
**L (Polymerase)**	RNA-dependent RNA polymerase; transcription and replication	Highly conserved enzymatic domains	Highly conserved enzymatic domains	~80% (aMPV/C vs. hMPV)	Strong conservation supports use in deep phylogenetic analyses

**Table 2:** Comparative Immunological Characteristics of aMPV and hMPV Infection.

Immune Feature	aMPV (Avian)	hMPV (Human)	Comparative Interpretation
**Innate immune activation**	Type I and III IFNs (IFN-α, IFN-λ); nitric oxide induction	Type I IFN responses are often dampened; altered TLR signaling	Both activate innate pathways, but hMPV more effectively suppresses antiviral signaling
**Key innate target cells**	Respiratory epithelial cells	Epithelial cells, alveolar macrophages, dendritic cells	Broader innate cell engagement in hMPV contributes to inflammation
**Cytokine profile**	IFN-γ, IL-6, IL-18 upregulated in respiratory tissues	Pro-inflammatory cytokines and chemokines drive disease severity	Overlapping inflammatory signatures with host-specific mediators
**T cell responses**	CD^4+^/CD^8+^ balance influenced by maternal antibodies	Protective immunity is incomplete; reinfections are common	Adaptive immunity is insufficient for sterilizing protection in both hosts
**Role of maternal antibodies**	Modulates T cell skewing and antibody induction	Passive maternal antibodies protect infants transiently	Early-life immunity shapes disease outcome and vaccine responses
**Humoral immunity**	IgA in mucosa; IgG systemically; incomplete protection	High seroprevalence by age 5; short-lived immunity	Antibody responses do not correlate fully with protection
**Immune evasion strategies**	Presumed targeting of avian innate pathways	Documented interference with STAT1, RIG-I signaling	Divergent immune evasion mechanisms adapted to host innate systems
**Contribution to pathology**	Immune-mediated epithelial damage; secondary infection risk	Immune-driven inflammation contributes to bronchiolitis and pneumonia	Immunopathology is a shared driver of clinical severity

**Table 3:** Comparative Tissue Tropism and Pathology of aMPV and hMPV.

Feature	aMPV (Avian Metapneumovirus)	hMPV (Human Metapneumovirus)	Comparative Interpretation
**Natural host range**	Domestic and wild birds (turkeys, chickens, others)	Humans (all ages), though it is most seen in children under 5 years old	Both viruses exhibit strict host specificity shaped by host-adapted entry and immune evasion strategies
**Primary target tissues**	Upper respiratory tract: nasal cavity, sinuses, trachea	Upper and lower respiratory tract	aMPV is largely restricted to the upper airways, while hMPV extends more consistently into the lower respiratory tissues
**Dominant target cell type**	Ciliated columnar epithelial cells	Ciliated airway epithelial cells; bronchiolar and alveolar epithelial cells	Shared tropism for ciliated epithelium reflects conserved replication niches
**Lower respiratory tract involvement**	Variable; often associated with co-infections	Frequent in infants, the elderly, and severe cases	Greater intrinsic lower airway tropism in hMPV
**Reproductive tract tropism**	Oviduct epithelium in laying birds	Not applicable	aMPV displays host-specific extrapulmonary tropism impacting egg production
**Gross pathology**	Facial swelling, conjunctivitis, sinusitis, swollen head syndrome	Bronchiolitis, pneumonia, and hypoxia in severe cases	Clinical pathology reflects anatomical and physiological host differences
**Histopathology**	Ciliostasis, epithelial necrosis, heterophil and mononuclear infiltration	Epithelial damage, inflammatory infiltrates, and alveolar involvement	Similar epithelial injury patterns with differing depth of tissue involvement
**Role of secondary infections**	Major driver of severity and mortality	Contributes to severity, especially in vulnerable populations	Both viruses predispose hosts to secondary infections, amplifying disease burden
**Experimental cross-species infection**	hMPV infects turkeys experimentally; no natural spillover	Limited spillover to non-human primates reported	Strong but incomplete host barriers exist under experimental conditions

**Table 4:** Vaccination strategy comparison between hMPV and aMPV.

Feature	hMPV (Human Metapneumovirus)	aMPV (Avian Metapneumovirus)	Comparative Interpretation
**Vaccine availability**	No licensed vaccines	Licensed vaccines are widely used in the poultry industry	Reflects differences in regulatory environment, host population, and economic drivers
**Predominant vaccine platforms**	Subunit, mRNA, live-attenuated (experimental/clinical)	Live-attenuated and inactivated (commercial)	aMPV relies on established whole-virus platforms; hMPV development emphasizes next-generation platforms
**Principal antigen target**	F glycoprotein (prefusion-stabilized)	Whole virus; F contributes but is not isolated as the sole antigen	Structural conservation of F drives hMPV design; aMPV vaccines prioritize broad exposure
**Role of prefusion F stabilization**	Central design principle informed by RSV experience	Not currently applied in commercial vaccines	Translation of RSV structural vaccinology primarily influences hMPV strategies, while traditional animal vaccines drive aMPV strategies
**Subunit vaccines**	Recombinant pre-fusion F and VLPs induce neutralizing antibodies	Not in current commercial use	Subunit approaches favored in humans for safety; limited uptake in poultry systems
**mRNA vaccines**	Actively in development; mRNA-1653 in clinical trials	Not available	Platform flexibility suits human vaccines, but not yet adapted to poultry production
**Live-attenuated vaccines**	Experimental; reverse genetics deletion of SH/G; intranasal delivery	Primary commercial strategy: field-derived attenuation	Shared goal of mimicking natural infection; scale and stability concerns differ
**Route of administration**	Intramuscular (subunit/mRNA); intranasal (live-attenuated)	Aerosol, spray, drinking water, oculonasal	Delivery reflects host logistics and the feasibility of mass vaccination
**Mucosal immunity induction**	Strong with live-attenuated candidates	Robust and rapid with live vaccines	Mucosal immunity is a shared priority for respiratory protection
**Safety considerations**	Risk of enhanced respiratory disease; infant safety critical	Reversion to virulence; vaccine strain shedding	Safety challenges are host- and platform-specific
**Evaluation of protection**	Neutralizing antibodies, durability, clinical outcomes	Reduction in clinical signs, shedding, mucosal, and cellular markers	Human vaccines emphasize correlates of protection; poultry vaccines emphasize functional outcomes
**Emerging optimization strategies**	Structure-guided antigen design; bivalent vaccines (hMPV/RSV)	Immunological biomarkers; schedule optimization; explant models	Innovation reflects distinct priorities: precision vs. operational effectiveness

## Data Availability

No new data was created or analyzed in this study. Data sharing is not applicable to this article.
